# Avian mortality risk during heat waves will increase greatly in arid Australia during the 21st century

**DOI:** 10.1093/conphys/coaa048

**Published:** 2020-09-08

**Authors:** Shannon R Conradie, Stephan M Woodborne, Blair O Wolf, Anaïs Pessato, Mylene M Mariette, Andrew E McKechnie

**Affiliations:** 1 South African Research Chair in Conservation Physiology, South African National Biodiversity Institute, 2 Cussonia Ave, Brummeria, Pretoria 0184, South Africa; 2 DST-NRF Centre of Excellence at the FitzPatrick Institute, Department of Zoology and Entomology, University of Pretoria, Lynnwood Rd., Pretoria 0002, South Africa; 3 iThemba LABS, Johannesburg, 514 Empire Rd, Johannesburg 2193, South Africa; 4 Mammal Research Institute, University of Pretoria, Lynnwood Rd., Pretoria 0002, South Africa; 5 UNM Biology Department, University of New Mexico, Albuquerque, NM 87131, U.S.A; 6 Centre for Integrative Ecology, School of Life & Environmental Sciences, Deakin University, 75 Pigdons Road, Waurn Ponds VIC 3216, Australia

**Keywords:** Avian mortality, dehydration, desert, heat waves, hyperthermia, population declines

## Abstract

Intense heat waves are occurring more frequently, with concomitant increases in the risk of catastrophic avian mortality events via lethal dehydration or hyperthermia. We quantified the risks of lethal hyperthermia and dehydration for 10 Australian arid-zone avifauna species during the 21st century, by synthesizing thermal physiology data on evaporative water losses and heat tolerance limits. We evaluated risks of lethal hyperthermia or exceedance of dehydration tolerance limits in the absence of drinking during the hottest part of the day under recent climatic conditions, compared to those predicted for the end of this century across Australia. Increases in mortality risk via lethal dehydration and hyperthermia vary among the species modelled here but will generally increase greatly, particularly in smaller species (~10–42 g) and those inhabiting the far western parts of the continent. By 2100 CE, zebra finches’ potential exposure to acute lethal dehydration risk will reach ~ 100 d y^−1^ in the far northwest of Australia and will exceed 20 d y^−1^ over > 50% of this species’ current range. Risks of dehydration and hyperthermia will remain much lower for large non-passerines such as crested pigeons. Risks of lethal hyperthermia will also increase substantially for smaller species, particularly if they are forced to visit exposed water sources at very high air temperatures to avoid dehydration. An analysis of atlas data for zebra finches suggests that population declines associated with very hot conditions are already occurring in the hottest areas. Our findings suggest that the likelihood of persistence within current species ranges, and the potential for range shifts, will become increasingly constrained by temperature and access to drinking water. Our model adds to an increasing body of literature suggesting that arid environments globally will experience considerable losses of avifauna and biodiversity under unmitigated climate change scenarios.

## Introduction

Understanding the effects of heat waves on species and their environments is becoming increasingly important as average temperatures and the likelihood of extreme heat anomalies and unpredictable rainfall increase as a result of anthropogenic climate change (IPCC, 2014; Ummenhofer and Meehl, 2017; Stillman, 2019). Multiple biological systems are already showing consequences of global heating, including mass mortalities (Welbergen *et al.*, 2014; Ratnayake *et al.*, 2019), extinctions (Pounds *et al.*, 1999; Thomas *et al.*, 2006) and rapid distribution shifts of species occuring in habitats ranging from polar to equatorial latitudes (Parmesan, 2006; Chen *et al.*, 2011).

Birds inhabiting environments where air temperatures (*T*_a_) frequently exceed normothermic body temperature (*T*_b_) experience particularly severe physiological challenges related to the avoidance of hyperthermia (Boyles *et al.*, 2011) and dehydration (Wolf and Walsberg, 1996) over time scales of minutes to hours (McKechnie and Wolf 2010; Albright *et al.*, 2017). When *T*_a_ > *T*_b_, small birds can lose >5% of their body mass (*M*_b_) per hour via evaporative cooling, even when inactive and in fully shaded microhabitats (Wolf and Walsberg, 1996; McKechnie and Wolf, 2010). Over longer time scales of days to weeks, chronic exposure to sustained hot weather has the potential to incur sublethal fitness costs associated with trade-offs between thermoregulation and foraging (du Plessis *et al.*, 2012; van de Ven *et al.*, 2019; Sharpe *et al.*, 2019). Similar trade-offs result in nest abandonment (Sharpe *et al.*, 2019) or reduced nestling provisioning on hot days influencing nestling growth rates, age at fledging and/or fledgling body mass, which ultimatly reduces reproductive success (Cunningham *et al.*, 2013; Wiley and Ridley, 2016; van de Ven *et al.*, in press). These sublethal fitness costs will likely drive large declines in arid-zone avian diversity under future climate change (Conradie *et al.*, 2019).

Under very hot conditions when birds can avoid lethal hyperthermia only via evaporative cooling, mortality risk can be predicted as the likelihood of lethal dehydration or lethal hyperthermia. Lethal dehydration risk in the absence of water intake during the hottest part of the day can be modelled as the probability of cumulative evaporative water loss (EWL) by resting birds exceeding their dehydration tolerance limits (McKechnie and Wolf, 2010; Albright *et al.*, 2017). The risk of lethal hyperthermia can be modelled as the probability of *T*_a_ exceeding species-specific heat tolerance limits (maximum *T*_a_ at which heat can be dissipated at a rate sufficient to defend *T*_b_ at sublethal levels; Whitfield *et al.*, 2015). Recent studies modelling the effect of global heating on EWL and survival for desert birds have revealed potentially devastating impacts on the survival of small desert species and important consequences for their water balance, daily activity and geographic distribution, particularly in the southwestern United States (Albright *et al.*, 2017). The avifauna of other regions, however, may experience far lower risks of lethal hyperthermia or dehydration effects of extreme heat (Conradie *et al.*, 2019), emphasizing the need for regional assessments of how the risks of acute heat exposure will change in coming decades.

Australia is characterized by a hot, arid climate and a terrestrial avifauna largely comprised of endemic taxa (Dingle, 2004; McKechnie *et al.*, 2012). Since 1910 average temperatures across the Australian continent have increased by just over 1°C (Hughes, 2003; Lewis and King, 2015). Rates of warming across most of Australia since 1951 are 0.1–0.2°C per decade, with the greatest rates of warming occurring inland and in south Western Australia (Collins *et al.*, 2000; Bradley *et al.*, 2003; Murphy and Timbal, 2008; Lewis and King, 2015). The frequency of extreme heat events has increased over recent decades (Collins *et al.*, 2000; Papalexiou *et al.*, 2018) with increasingly unpredictable rainfall events currently and under likely future conditions (IPCC 2014). Historical records of Australian climate and landbirds provide insight into the dramatic consequences that heat waves and drought can have on desert bird populations. For example, in January 2009 air temperatures > 45°C resulted in deaths of thousands of birds, including budgerigars and zebra finches in Western Australia (Towie, 2009). Other small endotherms have also been subject to heat-induced mortality events. For instance, the large pteropodid fruit bats of eastern Australia have experienced increasingly frequent mass mortality events (Welbergen *et al.*, 2008; Ratnayake *et al.*, 2019) with the most recent occurrence in November 2018 when approximately 23 000 grey-headed flying foxes (*Pteropus conspicillatus*) perished (Kim and Stephen, 2018). In light of these events, as well as the record-breaking heat wave of December 2019 (Bureau of Meteorology, 2020), assessing Australian species’ vulnerability to climate change based on their thermoregulation capacity limits is timely.

To quantify how the risks of lethal hyperthermia and lethal dehydration for the Australian arid-zone avifauna will change over the course of the 21st century, we synthesized recent thermal physiology literature data on evaporative cooling capacity and heat tolerance limits for Australian birds. We then evaluated risks of lethal dehydration in the absence of drinking during the hottest part of the day or of exceedance of heat tolerance limits under current climatic conditions compared to those anticipated by the end of this century. Assessing mortality risk in the absence of drinking during acute heat exposure allows inferences to be made about landscape suitability as a whole in terms of population viability, rather than restricting the risk assessment to small areas within close proximity to available surface water. This is particularly critical given the current and predicted severe reduction in water availability with unprecedented drought conditions. This assessment also provides a basis for evaluating the risks to Australian species associated with acute heat exposure compared to the avifaunas of two other regions, southern Africa (Conradie *et al.*, 2019) and southwestern United States (Albright *et al.*, 2017), in terms of frequency and geographic extent of such exposure. In addition, we asked whether the conditions currently experienced by Australian avifauna are novel relative to the recent past in terms of avian heat exposure and associated thermoregulatory demands. We addressed this question by mapping the spatiotemporal dynamics of *T*_a_ thresholds associated with lethal dehydration risk during the 20th and early 21st centuries.

## Materials and methods

### Species data

Data on thermal physiology are available for a number of Australian avian species inhabiting hot and arid regions across the continent. We restricted our analysis to recently published data collected using standardized methods involving exposure to stepped increments in *T*_a_ and very low chamber humidities (McKechnie *et al.*, 2016, 2017; Talbot *et al.*, 2017) for two reasons. First, unlike previous studies (e.g. Dawson and Bennett, 1973; Withers and Williams, 1990), these explicitly sought to elicit upper limits of heat tolerance and evaporative cooling capacity following the same protocol described by Whitfield *et al.* (2015). Second, earlier studies involved a range of chamber humidities that make direct comparisons among these studies difficult, as humidity exerts a strong influence on relationships between *T*_b_, EWL and RMR at *T*_a_ approaching and exceeding normothermic *T*_b_ (Gerson *et al.*, 2014; van Dyk *et al.*, 2019). For instance, the evaporative cooling capacity of spinifex pigeons, a classic arid-adapted Australian species, has been investigated only at *T*_a_ < 50°C at chamber relative humidities of 10–20% (Withers and Williams, 1990). However, recent data measured under lower humidity conditions for six columbids from three continents (McKechnie *et al.*, 2016; Smith *et al.*, 2015) has revealed heat tolerance limits of 56–62°C, raising the possibility that spinifex pigeons can tolerate environment temperatures well above 50°C.

In addition to these published data, we also included EWL measured at high air temperatures in zebra finches in a captive colony at Deakin University (Pessato and Mariette, unpublished data). Measurements of EWL involved a similar setup and experimental protocol to that described by Whitfield *et al.* (2015) and subsequently also used by McKechnie *et al.* (2016, 2017) and Talbot *et al.* (2017), but in this instance involved measurements at *T*_a_ up to 44°C (Pessato and Mariette, unpublished data). Preliminary EWL data for wild-caught zebra finches at *T*_a_ up to 46°C confirm that the dehydration threshold *T*_max_ value calculated from the data from the captive population is representative of wild populations (Pessato and Mariette, unpublished data). Heat tolerance limits were not elicited in this study, but Cade *et al.* (1965) reported zebra finches dying at *T*_a_ = 45–46°C, and preliminary data indicate that wild-caught zebra finches reach their thermal endpoint at *T*_a_ = ~ 46°C (Pessato and Mariette, unpublished data) and so we assumed a heat tolerance limit of 46°C for this species.

### Acute dehydration and hyperthermia risks

We used data on the relationship between *T*_a_, *T*_b_ and EWL to model acute heat exposure for 10 Australian species (Table 1). Risk of lethal hyperthermia was modelled as the daytime *T*_a_ exceeding species-specific heat tolerance limits reported in previous studies (Table 1). To model the risk of acute dehydration in the absence of drinking during the hottest part of the day, we adapted the methods described by Albright *et al.* (2017) and Conradie *et al.* (2019), where survival time was used to indicate the probability of death and/or the severity of a high-temperature event. Survival time was estimated as the number of hours during the hottest part of the day (starting at 10:00 hr) over which cumulative EWL remained below the equivalent of 15% of body mass (*M*_b_). This approach assumes that (i) birds are resting in deep shade during the hottest time of day, (ii) the operative temperature experienced by the bird is equivalent to air temperature and (iii) birds lose but do not gain water during this period of inactivity. Following the approach of Albright *et al.* (2017), we used an ecologically relevant survival time of ≤ 5 hr as a metric of moderate risk of lethal dehydration. Species-specific survival times under current conditions were estimated using an average diurnal temperature profile calculated from the 10 hottest days during the past decade (2000–2010 CE) across Australia, and future survival times using the same curve shifted upwards to account for the predicted increases in maximum daily air temperature (*T*_max_ = 4–5°C, RCP 8.5).

**Table 1:** Parameters used for modelling acute heat exposure in Australian arid-zone birds

Species	Mass (g)	EWL ~*T*_a_	Dehydration *T*_max_ (°C)	HTL (°C)	Source
Zebra finch (*Taeniopygia guttata*)	12	*0.08T_a_–1.58	41.5	46	Pessato and Mariette (unpublished data)
Yellow-plumed Honeyeater (*Lichenostomus ornatus*)	17	0.09T_a_–3.29	42.5	46	McKechnie *et al.* (2017)
Spiny-cheeked honeyeater (*Acanthagenys rufogularis*)	42	0.18T_a_–6.65	44	48	McKechnie *et al.* (2017)
Chestnut-crowned babbler (*Pomatostomus ruficeps*)	53	0.23T_a_–8.85	45.5	48	McKechnie *et al.* (2017)
Grey butcherbird (*Cracticus torquatus*)	86	0.32T_a_–11.95	45.5	50	McKechnie *et al.* (2017)
Apostlebird (*Struthidea cinerea*)	118	0.30T_a_–10.80	47	52	McKechnie *et al.* (2017)
Mulga parrot (*Psephotellus varius*)	55	0.23T_a_–8.66	45	44–49	McWhorter *et al.* (2018)
Galah (*Eolophus roseicapilla*)	265	0.13T_a_–2.81^a^; 0.66T_a_−24.36^b^	45	53–55	McWhorter *et al.* (2018)
Australian owlet-nightjar (*Aegotheles cristatus*)	44	0.21T_a_–8.5	46.5	52	Talbot *et al.* (2017)
Crested pigeon (*Ocyphaps lophotes*)	186	0.268T_a_–9.647	54.6	>60	McKechnie *et al.* (2016)

^*^Whole-animal values in mg H_2_O min^−1^

Body masses and the relationship between EWL and air temperature (*T*_a_) were obtained from the sources listed. Estimates of EWL as a function of *T*_a_ refer to whole-animal values in g H_2_O hr^−1^; these relationships were used to calculate cumulative EWL from daily *T*_a_ traces. Also shown are the maximum daily air temperature (*T*_max_) associated with moderate risk of lethal dehydration; these were calculated by modelling EWL during the course of a hot day. For the latter calculations, we generated an average daily *T*_a_ profile using data from the hottest day in each year between 2000 and 2010

^a^Evaporative water loss at 30 °C < T_a_ < 41.3 °C. Galahs have two significant inflection points characterizing the relationship between EWL and T_a_, where an upper inflection exists at 41.3 °C

^b^T_a_ < 41.3 °C

Water present in the crop when birds cease activity or metabolic water produced during the digestion of food while inactive could potentially extend the time taken for cumulative EWLs to reach dehydration tolerance limits. To evaluate the sensitivity of our model to the assumption of zero water gain during the period of inactivity, we included an additional analysis of water-in-crop dehydration risk under current conditions for fully hydrated zebra finches using the maximum mass of crop contents (~0.5 g) as additional water available for evaporative cooling (Meijer *et al.*, 1996).

### Climate models and analyses

The methods described by Conradie *et al.* (2019) were used to model current and future climate conditions across Australia. In brief, data for modern climatic conditions (1850–2014) were obtained from the Physical Sciences Division of the National Oceanic and Atmospheric Administration’s Earth Systems Research Laboratory (Boulder, CO; https://www.esrl.noaa.gov/pds/). We selected modelled climate data from NOAA Cooperative Institute for Research in Environmental Sciences 20th Century Reanalyses (v2c), where forcing fields were interpolated to 1.88° latitude × 1.88° longitude. We restricted the temporal resolution to daytime values only (06:00–18:00) over the austral summer season (October–March). Experiment r6i1p1 and RCP 8.5 scenario of the CCSM4 projection from CMIP V (https://cmip.llnl.gov/cmip5/) was used for the future (2076–2100 CE) climate change scenario. These projections were obtained from the National Center for Atmospheric Research (Boulder, CO; https://esgf-node.ipsl.upmc.fr/search/cmip5-ipsl/), with forcing fields interpolated to 0.95° latitude × 1.25° longitude. This model forms part of a collaborative effort demonstrating strong model convergence, has been parameterized for biological research and is reflective of multimodel future climate change predictions (Flato *et al.*, 2013; IPCC, 2014). Additionally, climate models projecting the greatest warming for the end of the century are increasingly found to the models best simulating current conditions (Zhai *et al.*, 2015; Brient and Schneider, 2016; Brown and Caldeira, 2017). We used a business-as-usual, unmitigated climate change projection (RCP 8.5) for 2100 CE as the most likely scenario (SI Appendix, RCP 4.5). Distribution data were obtained from BirdLife International and NatureServe (2013) (http://datazone.birdlife.org/species/search) for all 10 bird species. These data were compiled from several sources, including museum records, observation and occurrence data, distribution atlases and maps from surveys and field guides. Data on reported sightings were obtained from Birdata (https://birdata.birdlife.org.au/). All data analyses were conducted in the R programming environment (R Core Team, 2015) using the R Studio (version 3.2.3) interface.

## Results

### Interspecific variation in current and future risks

**Figure 1:**
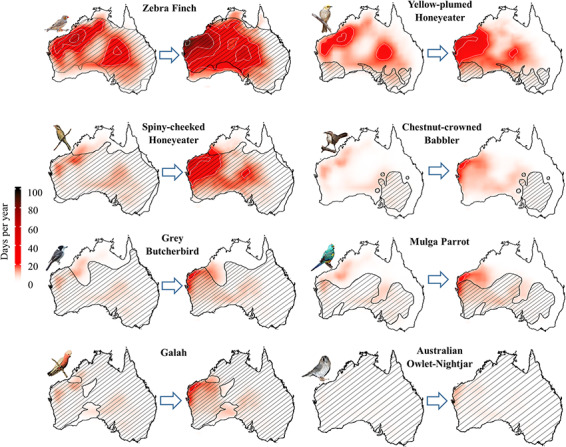
Average number of days per year with conditions associated with a moderate lethal dehydration risk (i.e. survival time <5 hr) across Australia for eight species under recent (left, 2000–2010 CE) and future conditions (right, 2080–2090 CE) assuming a business-as-usual future emissions scenario (RCP 8.5). Species ranges illustrated by cross-hatching. Bird images courtesy of the Macaulay Library at the Cornell Lab of Ornithology [Photographers: Chris Wiley (ML38358341, ML86083871, ML146062151, ML113207541), Rufus Wareham (ML108255531), Indra Bone (ML116328301), Andrew Allen (ML37228621), Stephen Murray (ML74723661)].

**Figure 2:**
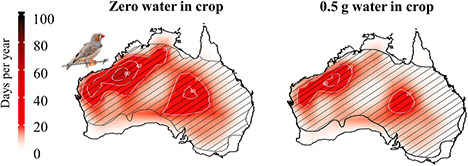
Average number of days per year with conditions associated with a moderate lethal dehydration risk (i.e. survival time <5 hr) for zebra finches (*T. guttata*) across Australia under recent climatic conditions (2000–2010 CE) for birds that are fully hydrated with no water or food stored in their crop at the onset of the period of inactivity on a very hot day (left) and birds whose crops are full of water (right). Species ranges illustrated by cross-hatching. Bird images courtesy of the Macaulay Library at the Cornell Lab of Ornithology [Photographer: Chris Wiley (ML38358341)].

The 10 species we modelled here vary widely in their exposure to conditions associated with lethal dehydration under both current and future conditions. Under recent (2000–2010) conditions and assuming no drinking during the hottest part of the day, zebra finches, yellow-plumed honeyeaters and spiny-cheeked honeyeaters experienced a meaningful risk of lethal dehydration on > 15 d y^−1^ in some parts of their ranges, mainly centred on northwestern Australia with up to 28 d y^−1^ experienced by zebra finches (averaged over S 18° 6′–S 25° 43′ and E 114° 23′–E 125° 36′; Fig. 1). Larger species experienced lower risks, ranging from < 10 d y^−1^ in chestnut-crowned babblers and grey butcherbirds to zero d y^−1^ in apostlebirds and crested pigeons. By the 2080s, these risks will expand greatly for all modelled species, with the exception of chestnut-crowned babblers, apostlebirds and crested pigeons. Increases in the frequency and spatial extent of lethal dehydration risk in the absence of water intake will be greatest for smaller species, in particular zebra finches and spiny-cheeked honeyeaters (Fig. 1; ESM Fig. S1). In the case of zebra finches, exposure to lethal dehydration will reach ~ 100 d y^−1^ in the far northwest and will exceed 20 d y^−1^ over > 50% of this species’ range (Fig. 1). Adjustments for a water-in-crop scenario for this species resulted in only a 1°C increase in the maximum *T*_a_ predicted as the threshold for dehydration. Exposure to lethal dehydration for fully hydrated zebra finches will occur over an ~30% smaller area and with a lower frequency than birds with zero water (40–60 d y^−1^; Fig. 2). Similar increases will occur for spiny-cheeked honeyeaters, whereas for larger species these increases will be more modest and largely restricted to the north and northwestern regions of Western Australia. For instance, grey butcherbirds will experience an increase from ~ 2.3 d y^−1^ currently to ~ 9.7 d y^−1^ by the end of the century (averaged over S 18° 6′–S 25° 43′ and E 114° 23′–E 125° 36′).

**Figure 3:**
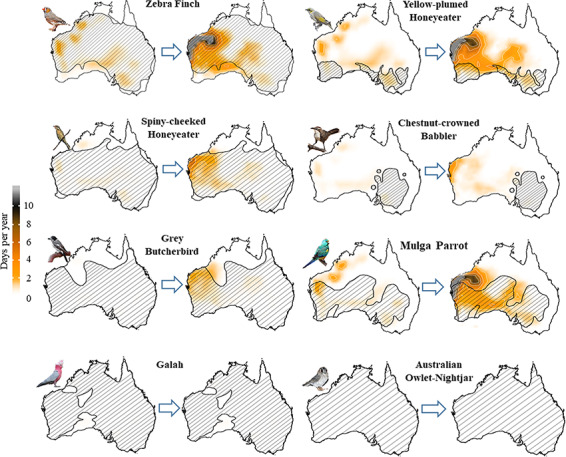
Average number of days per year with conditions associated with lethal hyperthermia risk (i.e. air temperature exceeding species-specific heat tolerance limits) across Australia for eight species under recent (left, 2000–2010 CE) and future conditions (right, 2080–2090 CE) assuming a business-as-usual future emissions scenario (RCP 8.5). Grey regions indicate exposure > 10 d y^−1^ but not exceeding 20 d y^−1^. Species ranges illustrated by cross-hatching. Bird images courtesy of the Macaulay Library at the Cornell Lab of Ornithology [Photographers: Chris Wiley (ML38358341, ML86083871, ML146062151, ML113207541), Rufus Wareham (ML108255531), Indra Bone (ML116328301), Andrew Allen (ML37228621), Stephen Murray (ML74723661)].

Lethal hyperthermia risks under current conditions are lower than dehydration risks, with zebra finches, yellow-plumed honeyeaters, spiny-cheeked honeyeaters and mulga parrots (HTL = 44–49°C; Table 1) all experiencing < 10 d y^−1^ exposure to lethal hyperthermia risk currently over limited parts of their ranges (Fig. 3 & ESM Fig. S2). By the end of the century, these conditions are predicted to increase in frequency (more days per year) and geographic area. For spiny-cheeked honeyeaters, exposure to lethal hyperthermia will increase from ~ 1 d y^−1^ to > 6 d y^−1^ in the far north region of Western Australia (Fig. 3). Consistent with lethal dehydration risk, smaller species will experience the greatest increase in both the frequency and spatial extent of lethal hyperthermia risk. In particular, the mulga parrot (M_b_ = 55 g) will experience ~ 20 d y^−1^ in the far northwestern edge of their range and will exceed 5 d y^−1^ over approximately one third of their range (ESM Fig. S2). The remainder of the species (apostlebird, galah, Australian owlet-nightjar and crested pigeon) are not likely to experience environmental conditions where their HTLs are exceeded by the end of this century (Table 1; Fig. 3; ESM Fig. S2).

### Novelty of recent conditions in terms of avian heat exposure

**Figure 4:**
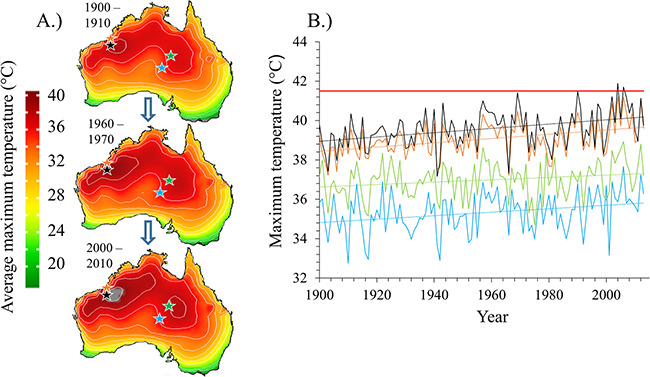
Spatial-temporal representation of the average maximum temperatures (°C) across Australia depicted spatially throughout the past 100 years, with grey indicating areas where *T*_a_ exceeds threshold values associated with moderate lethal dehydration risk (A). Time-series extractions from the four hottest locations identified in the spatial-temporal analyses, where the red line indicates the lowest threshold value likely to induce lethal dehydration (B). A linear regression line was fitted for each location to illustrate the change in temperatures over time.

**Figure 5:**
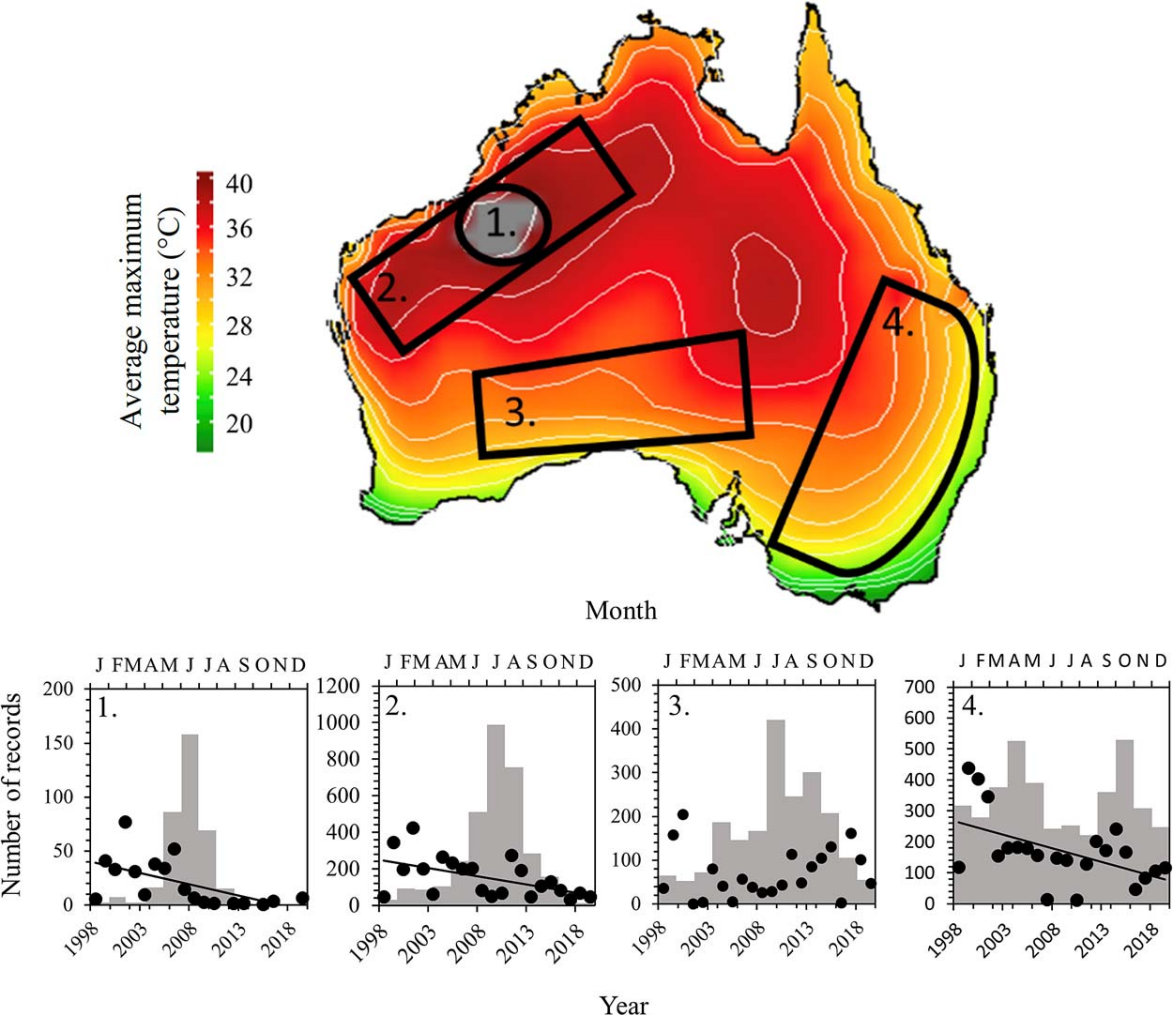
Analyses of atlas records for zebra finches (*T. guttata*) in four regions varying in average air temperature maxima between 2000 and 2010. The grey area indicates average air temperature maxima > 40°C. The panel for each area shows total records per year (filled circles, year on lower *x*-axis) as well as the total seasonal distribution of records per month summed over the 20 year period (grey histogram; months on upper *x*-axis). Both records per year and seasonal records are reported on the *y*-axis. The solid lines represent significant linear regression models fitted to the yearly data (*P* < 0.05).

An analysis of average summer maximum air temperatures across the Australian continent confirms that birds are currently experiencing conditions unlike those that prevailed over the course of the 20th century (Fig. 4). None of the study species were exposed to average summer *T*_a_ maxima associated with lethal dehydration risk during the 20th century, whereas all modelled species (except apostlebirds and crested pigeons) now experience some risk on a small number of days per year over limited parts of their ranges (Fig. 1). In the case of zebra finches, for which our EWL data reveal that daily maximum *T*_a_ > 41.5°C is associated with risk of lethal dehydration, maximum summer average *T*_a_ has recently begun to exceed this threshold in some parts of northwestern Australia (Fig. 4). Reported sightings of zebra finches during recent years have declined across Australia, most noticeably in areas where average maximum temperatures have exceeded 41.5°C (Fig. 5).

## Discussion

The climatic conditions currently experienced by Australian birds are unlike those of the 20th century, and our analyses reveal that several species already encounter conditions associated with significant risks of lethal hyperthermia or dehydration. In the absence of meaningful reductions in greenhouse gas emissions, the likelihood of avian mortality during heat waves will increase substantially over much of the continent by the end of the 21st century. Increased water demands for evaporative cooling will be a major mechanism driving elevated mortality risk unless birds have access to drinking water during the hottest part of the day, but several species will also be at substantial risk of lethal hyperthermia resulting from air temperatures exceeding their heat tolerance limits. Overall, the combination of increasing temperatures and unpredictable water availability under climate change poses a significant risk to the small-sized avifauna of the Australian interior.

### Lethal dehydration and hyperthermia risks

Environmental conditions associated with the risk of lethal dehydration have not, until recently, been routinely experienced by Australian arid-zone birds, with *T*_max_ seldom exceeding threshold values for a risk of lethal dehydration in the absence of drinking during the hottest part of the day. Among the species for which we have modelled this risk, only zebra finches likely experienced temperatures approaching values associated with lethal dehydration risk over large parts of their range during the last century (Fig. 4). However, in recent decades, temperatures have increased to levels that pose a meaningful risk of lethal dehydration for most species modelled here over at least some parts of their ranges.

Of the species modelled here, zebra finches emerged as the most susceptible to lethal dehydration risk under past, recent and future conditions. Modifying the lethal dehydration risk model for this species to account for water storage in the crop did not markedly alter our conclusions. Our EWL data for this species are very similar to those collected by Calder and King (1963), with the *T*_max_ value predicted to be the threshold for lethal dehydration risk differing by only 0.5°C between the two data sets. Moreover, our threshold *T*_max_ for lethal dehydration (*T*_max_ = 41.5°C) falls well within the range recently estimated for small, regularly drinking passerines from the southern African arid zone (37–44°C; Czenze *et al.*, in press). This relatively low *T*_max_ threshold for zebra finches, combined with the extent of this species’ regular exposure to *T*_max_ approaching or exceeding 40°C (Fig. 4), suggests it must continue drinking during the hottest part of the day to survive high temperatures. This prediction is supported by recent observations of zebra finches with access to artificial feeders and water troughs in close proximity to breeding colonies, where the birds continued to visit water stations on very hot afternoons (Cooper *et al.*, 2019). Under these conditions, individuals were able to maintain water and energy balance at *T*_max_ up to 44.5°C, over a 1–3 d period (Cooper *et al.*, 2019). This observation for zebra finch activity is broadly consistent with a recent analysis revealing that the cessation of activity during hot weather occurs at significantly higher *T*_a_ among Australian compared to southern African arid-zone birds (Pattinson *et al.*, 2020).

When considered independently, the risk of lethal hyperthermia faced by Australian birds remains lower than that of lethal dehydration under both recent and future climate conditions. Among the passerine species for which heat tolerance data are available, HTL generally increases with body mass (McKechnie *et al.*, 2017), with the result that smaller species may be more vulnerable to both dehydration and hyperthermia during extreme heat events, at least among the species we have modelled. To the best of our knowledge, this is the first study to predict, on the basis of species-specific empirical data, mortality as a direct consequence of birds being physiologically incapable of maintaining *T*_b_ below *T*_a_ on very hot days. Although our species-specific *T*_a_ thresholds for hyperthermia risk are generally higher than the corresponding thresholds for dehydration risk (Table 1), the former thresholds are based on cumulative water losses over several hours of heat exposure. In contrast, lethal hyperthermia can result from even relatively brief exposure to *T*_a_ exceeding HTL, and so during extreme heat events mortality from *T*_b_ reaching lethal levels may well precede cumulative water losses reaching lethal levels. The risks of lethal hyperthermia we document here emphasize the need to consider this potential source of mortality for arid-zone birds in addition to increased evaporative cooling requirements (e.g. Riddell *et al.*, 2019). That many Australian birds, particularly passerines, face a substantial risk of lethal hyperthermia under future conditions is not unexpected in view of the generally lower HTL values of Australian passerines compared to those from southern Africa and the American southwest (Whitfield, *et al.*, 2015; McKechnie *et al.*, 2017; Smith *et al.*, 2017).

Risks of lethal dehydration and lethal hyperthermia are, however, likely not independent of each other. For the species modelled here, threshold *T*_a_ values for lethal hyperthermia are higher than threshold *T*_max_ values for lethal dehydration, as is also the case for 12 southern African arid-zone passerines, with the difference between these thresholds larger for regularly-drinking, water-dependent species (Czenze *et al.*, in press). Taken together with Cooper *et al*.’s (2019) observations of zebra finches drinking at *T*_a_ as high as 44.5°C when water is available nearby, this suggests a scenario wherein birds continue visiting water to drink when *T*_a_ is above thresholds associated with lethal hyperthermia. However, this behaviour could substantially increase lethal hyperthermia risk because (i) water sources are often in unshaded locations where solar heat gain results in operative temperature (*T*_e_) exceeding *T*_a_ by 10–15°C in small species (Bakken, 1976; Robinson *et al.*, 1976; Wolf and Walsberg, 1996; Wolf, 2000) and (ii) moving from shaded microsites to water which may be several kilometres away results in an additional metabolic heat load as a by-product of activity. For species that rely on drinking during extremely hot conditions, lethal hyperthermia may represent a much greater source of mortality than lethal dehydration *per se*.

Trade-offs between managing the risk of lethal dehydration and lethal hyperthermia in small birds may lead to currently occupied habitats away from water sources becoming inhospitable, as the required frequency of drinking throughout the day increases with concomitant increases in temperature. Furthermore, recent observations and projected rainfall conditions indicate that water availability in arid Australia will likely decrease severely under climate change, including at artificial water points (Delworth and Zeng, 2014). Most strikingly, many dams and bores throughout extensive parts of the Australian arid and semi-arid zone that had so far been considered as permanent water points (including during normal droughts in the typical boom and bust cycle of inland Australia) dried out over the 2018–2019 period (Bureau of Meteorology, 2019), with natural water points likely faring worse. Therefore, unlike current practices in conservation areas decommissioning artificial water sources, maintaining water availability may become a necessary conservation practice to mitigate the impact of climate change on small bird communities.

Recent and historical accounts of mass mortality events involving Australian avifauna provide anecdotal support for the idea that lethal hyperthermia may be the major source of mortality. Zebra finches featured prominently in historical accounts of mass mortality during heat waves (Finlayson, 1932), and Bech and Midtgård (1981) argued that this species is particularly sensitive to hyperthermia because it lacks a well-developed *rete mirabile ophthalmicum* to facilitate brain cooling. Several photographs from an event involving budgerigars (*Melopsittacus undulatus*) in Western Australia in January 2009 (Towie, 2009) show dead birds around a pool of water. In an account of similar avian mortality at a railway station in January 1932, Finlayson (1932) attributed most deaths to hyperthermia, noting that only a small fraction of the hundreds of severely heat-stressed birds were attempting to drink from water provided by railway staff. Other accounts of the same heat wave that struck a vast region of south-central Australia documented tens of thousands of dead birds in and around water tanks and troughs (McGilp, 1932), suggesting that lethal hyperthermia, arising in part from metabolic heat produced while flying to water, was the proximate cause of mortality

The increasing frequency and geographical extent of risks of heat-related mortality, whether arising from lethal dehydration or hyperthermia, suggest that the ranges of arid-zone species will contract out of very hot areas. To evaluate this possibility, we examined atlas data for zebra finches obtained from https://birdata.birdlife.org.au/. Reporting rates suggest that this partly nomadic species (Zann, 1996) is largely absent in summer from the region in northwestern Australia where average summer *T*_max_ approaches the threshold for lethal dehydration risk on hot days, and almost entirely absent during summer from the area of the Great Sandy Desert over which average conditions already exceed this threshold (Fig. 5, panels 2 and 1, respectively). The seasonal variation in reporting rates is more pronounced in these regions compared to cooler parts of the continent, particularly the more mesic east (Fig. 5). Trends in reporting rates over the past two decades also suggest that proportionately greater declines occur in these very hot areas compared to elsewhere (Fig. 5). Whereas these patterns in reporting rates could reflect other factors, particularly given the remoteness of some of the areas involved, it is nevertheless striking that they match very closely those predicted by our model of dehydration risks associated with acute heat exposure. The exact mechanisms driving these declines need further investigation and may be due to a combination of factors including reduced breeding success, nestling provisioning and survival in areas associated with prolonged high average temperatures.

Threshold *T*_a_ values for lethal dehydration and lethal hypothermia in crested pigeons are substantially higher than those of other species modelled (Table 1), reflecting the large *M*_b_ of this species and columbids’ capacity for rapid and efficient cutaneous evaporation (McKechnie *et al.*, 2016). An increase in this species’ abundance has been reported at a long-term monitoring site in mesic eastern Australia (Greenville *et al.*, 2018), supporting the notion that columbids are less likely to be negatively affected than many of the species modelled here as long as they have access to daily drinking water.

### Assumptions and limitations

One of the key assumptions of our analysis is that the relationships between *T*_a_ and *T*_b_ observed under laboratory conditions are representative of free-ranging birds under natural conditions. The limited available literature on hyperthermia in free-ranging birds suggests that *T*_b_ values observed under laboratory conditions are similar to those of free-ranging birds (Whitfield *et al.*, 2015; McKechnie *et al.*, 2016; Thompson *et al.*, 2018). Furthermore, our model assumes that *T*_a_ approximates the *T*_e_ actually experienced by birds roosting in fully shaded microsites. Many shaded sites available to birds will involve *T*_e_ > *T*_a_ on account of vegetation characteristics and factors such as radiative heat gains from the ground, although in some instances might be slightly cooler than *T*_a_, for example, in microsites provided by mistletoes, where high rates of transpiration can result in *T*_a_ below that of the surroundings (Little *et al.*, 2012) or under trees with large trunks and canopies and slower heating rates (Wolf and Walsberg, 1996). On the other hand, some bird species nest in full sunlight (Grant, 1982; Walsberg and Voss-Roberts 1983; Tieleman *et al.*, 2008), where an incubating bird can experience *T*_e_ ≥ 15°C higher than instrumental *T*_a_ (O’Connor *et al.*, 2018).

Phenotypic plasticity in physiological traits related to heat tolerance via acclimation or acclimatization has the potential to alter temperature thresholds for hyperthermia and dehydration (Marder and Arieli, 1988; McKechnie and Wolf, 2004; Noakes *et al.*, 2016). Our understanding of the physiological mechanisms responsible for intraspecific variation in avian thermoregulation to high *T*_a_ is limited, but recent work has uncovered a novel mechanism of developmental plasticity in zebra finches (Mariette and Buchanan, 2016), suggesting a potential for the development of more heat tolerant phenotypes via developmental plasticity and epigenetic transmission of traits. However, given the extent and rapid advancement of climate change, the overall avian capacity to adapt physiology and behaviour may be limited (Collins *et al.*, 2000; Parmesan and Yohe, 2003; Papalexiou *et al.*, 2018).

Finally, our analysis is based on a single future climate change projection from the Coupled Model Intercomparison Project (CMIP), whereas other simulations may predict different temperature changes and climate trajectories across Australia. The models which comprise the CMIP initiative have strong convergence between them, suggesting that although absolute values may differ between projections the overall trend is likely to follow the scenarios on which our modelling is based. Our selection of the RCP 8.5 scenario of the CCSM4 projection appears well supported based on the current increases in atmospheric CO_2_ and may actually represent a conservative estimate based on positive feedbacks in the climate system that may lead to increased rates of warming (Turetsky *et al.*, 2019). Therefore, we are confident that our major conclusion of increased heat exposure in coming decades will not change using different future climate models.

### Global variation in risks of lethal effects of acute heat exposure

The projected exposure of Australian species to lethal dehydration and hyperthermia risk reiterates that the magnitude of these risks varies considerably among regions. Small passerines such as zebra finches and spiny-cheeked honeyeaters will routinely experience costly heat exposure for > 40 d per summer in the western parts of their ranges and > 60 d per summer in Australia’s far northwest, levels of exposure quantitatively similar to those projected for passerines inhabiting the deserts of southwest North America (Albright *et al.*, 2017). These authors predicted that all five passerines modelled will be exposed to at least 1 d per year of survival time <5 hr over 50% or more of their range by the end of the 21st century. In southwestern Arizona, species such as the lesser goldfinch, cactus wren and curve-billed thrasher will regularly experience lethal dehydration risk for ≥50 d per year (Albright *et al.*, 2017). Levels of risk for Australian and North American species are far greater than those for birds in southern Africa’s Kalahari Desert, where the risk of acute heat exposure will remain relatively low (<10 d per year) in coming decades (Conradie *et al.*, 2019), largely on account of the lower current and predicted future *T*_max_ values in the southern African arid zone.

Species’ movement ecology is likely to be an important determinant of how they respond to increased risks of lethal effects of acute heat exposure. The high degree of nomadism among the Australian avifauna, with 30–46% of inland breeding bird species considered to be nomads or partial nomads (Dean, 2004), may provide a potential buffer from heat-driven mortality in many species. The prevalence of nomadism in Australia has been linked to the continent’s highly variable rainfall (Wiens, 1991), with most studies focusing on rainfall and food availability as primary drivers of nomadism (e.g. Allen and Saunders, 2002; Tischler *et al.*, 2013; Smith, 2015). Under current and future climates, however, temperature and water availability may emerge as a much stronger driver of nomadic movements than has been the case in the past. However, the Australian heat wave of late 2019 reiterates that nomadism may fail to prevent many populations from being `trapped’ by extreme high temperature events given the broad geographic extent of current heat events. During December 2019, maximum *T*_a_ averaged > 42°C over large areas of the Australian interior and most of the continent experienced maxima > 45°C (Bureau of Meteorology, 2020). In late December 2019, heat-related mortalities were recorded at Gluepot Reserve (S 33°46′, E 140°07′), the site where most of the physiological data on which this analysis is based were collected (Johnston, *pers. comm*.; Gluepot Reserve, 2019).

In addition to increased direct mortality risk, higher temperatures also increase exposure to sublethal costs of sustained hot weather affecting various components of fitness including adult body condition (du Plessis *et al.*, 2012; Andrew *et al.*, 2017; van de Ven *et al.*, 2019; Sharpe *et al.*, 2019) and breeding success (Cunningham *et al.*, 2013; Wiley and Ridley, 2016). In southern Africa, the long-term effects of trade-offs in thermoregulatory behaviour are likely to affect the persistence of avian populations even in regions where temperatures do not exceed threshold values likely to induce mass mortality events (Conradie *et al.*, 2019). In North America and Australia, several studies have reported similar effects of high temperatures on body condition (Carroll *et al.*, 2015; Cruz-Mcdonnell and Wolf, 2016; Edwards *et al.*, 2015) but little is known about the magnitude of these effects. Sharpe *et al.* (2019) recently observed wholesale nest abandonment and a three-fold increase in adult mortality in a population of jacky winters (*Microeca fascinans*) during extreme heat waves in southern Australia during 2018–2019. In another study, exposure to increased temperatures and prolonged drought resulted in a 98% decline in breeding pairs of burrowing owls in southwestern United States between 1998–2013 (Cruz-McDonnell and Wolf, 2016). These sublethal fitness consequences suggest, contrary to the conclusions of Riddell *et al.* (2019), that cooling costs are not the only determinant of species declines currently or under future climate change scenarios.

## Conclusions

We have presented a baseline model of the acute, lethal risks posed by global heating to a subset of Australian birds. The risks of mass mortality via lethal dehydration and hyperthermia for the species modelled here will increase in severity, frequency and geographic extent in a manner quantitatively similar to that anticipated for desert birds in the southwestern United States (Albright *et al.*, 2017). The general lack of exposure of Australian birds to conditions associated with risks of lethal dehydration or hyperthermia during the 20th century provides a new perspective on the catastrophic mortality that occurred over large parts of central Australia in January 1932 (Finlayson, 1932). Our historical analysis of risks of heat-associated mortality supports the notion that the die-offs reported on an unprecedented scale in 1932 (Finlayson, 1932; Serventy, 1971) were in fact highly unusual events for the 20th century, but also reveals that the likelihood of events on a similar scale has increased in recent decades and will greatly increase further. Moreover, the likelihood of persistence within and the movement out of modelled species ranges may become increasingly constrained by temperature, with the exception of species with ranges restricted to the eastern parts of Australia such as apostlebirds and chestnut-crowned babblers, which will remain at relatively low risk of heat exposure. We have provided an analysis for a subset of avian species for which physiological data are available, but which are likely indicative of the consequences of increasingly hot conditions for the majority of arid-zone avian species. Although pteropodid bats have been the most prominent victims of extreme heat in the first two decades of the 21st century (Welbergen *et al.*, 2008; Shea *et al.*, 2016), we predict that avian die-offs will be reported much more frequently in the future. This prediction is supported by several news and social media reports of avian mortality during the record-breaking heat wave experienced by Australia in December 2019 (Gluepot Reserve, 2019; Rolfe, 2019). Given the combined effects of high *T*_a_ values, our model suggests that arid environments globally will experience considerable losses to avifauna and biodiversity under unmitigated climate change scenarios. Conservation strategies ensuring the maintenance of surface water availability throughout landscapes in spite of increasingly severe droughts and land-use changes might prove crucial for preventing large areas of the arid zone becoming uninhabitable for many avian species.

## Funding

This work was supported in part by the National Research Foundation of South Africa (Grant Number 119754 to A.E.M.). Any opinions, findings and conclusions or recommendations expressed in this material are those of the author(s) and do not necessarily reflect the views of the National Science Foundation (grant IOS 1122228 to B.O.W.) or the National Research Foundation of South Africa.

## Supplementary Material

Australia_MS_ESM_coaa048
