# Supplementary material for: Avian mortality risk during heat waves will increase greatly in arid Australia during the 21st century
Source: Conserv Physiol. 2020 Sep 8;8(1):coaa048. doi: 10.1093/conphys/coaa048 (PMC7271765; doi:10.1093/conphys/coaa048)
Supplement: Australia_MS_ESM_coaa048 [file australia_ms_esm_coaa048.pdf]

ESM - Avian mortality during heat waves will increase greatly in arid Australia during the 21st Century

Figure legends

Figure S1. Average number of days per year with conditions associated with a moderate dehydration risk (i.e. survival time < 5 hours) across Australia for eight species under recent (2000 – 2010 CE) and future conditions (2080 – 2090 CE) assuming a moderate future emissions scenario (RCP 4.5). Species ranges illustrated by cross-hatching.

Figure S2. Average number of days per year with conditions associated with a moderate hyperthermia risk (i.e. air temperature exceeding species-specific heat tolerance limits) across Australia for seven species under recent (2000 – 2010 CE) and future conditions (2080 – 2090 CE) assuming a moderate future emissions scenario (RCP 4.5). Grey regions indicate exposure > 10 d y^{-1} but not exceeding than 20 d y^{-1}. Species ranges illustrated by cross-hatching.

Figure S1

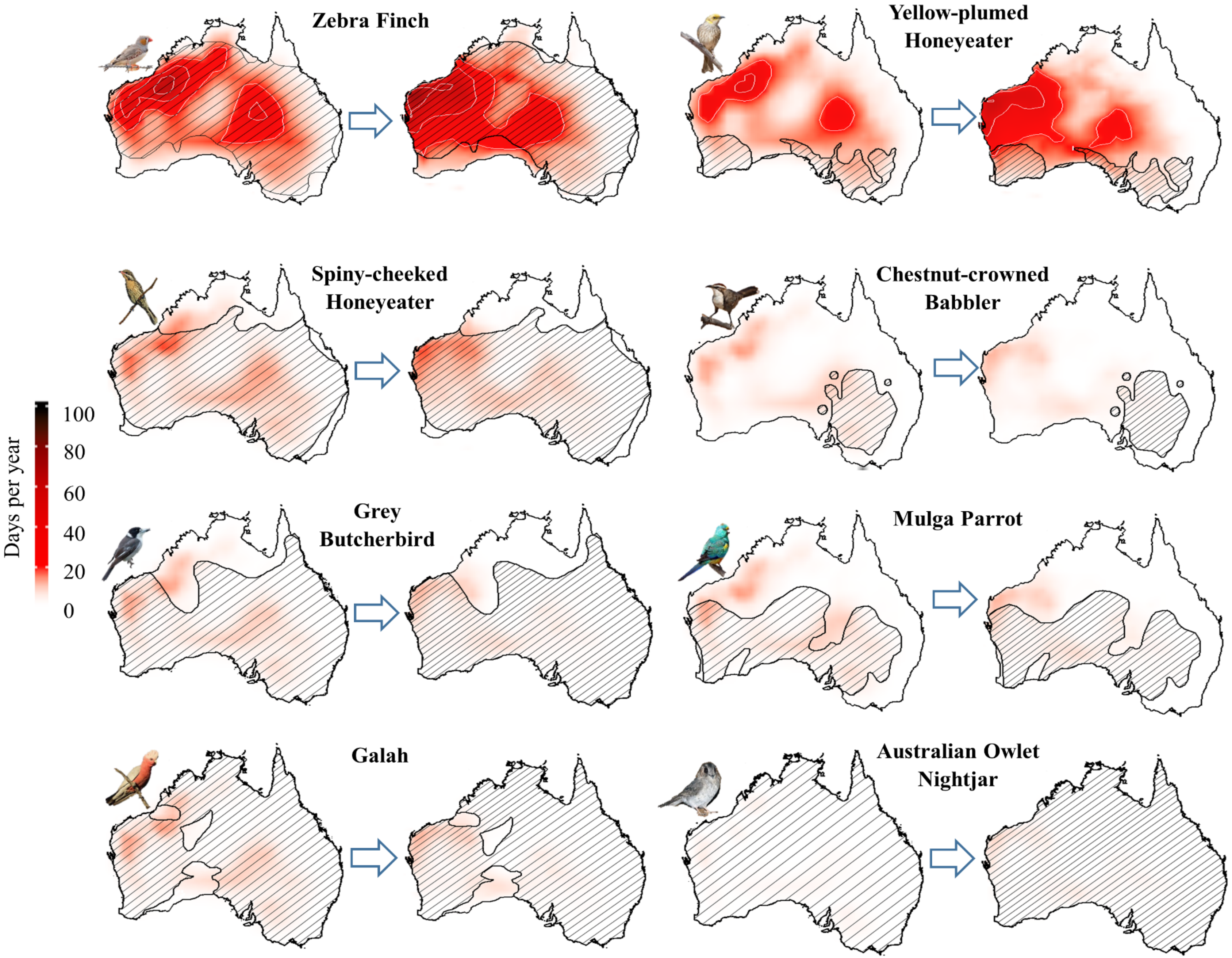
Zebra Finch
Yellow-plumed Honeyeater
Spiny-cheeked Honeyeater
Chestnut-crowned Babbler
Days per year
100
80
60
40
20
0
Grey Butcherbird
Mulga Parrot
Galah
Australian Owlet Nightjar

Figure S2

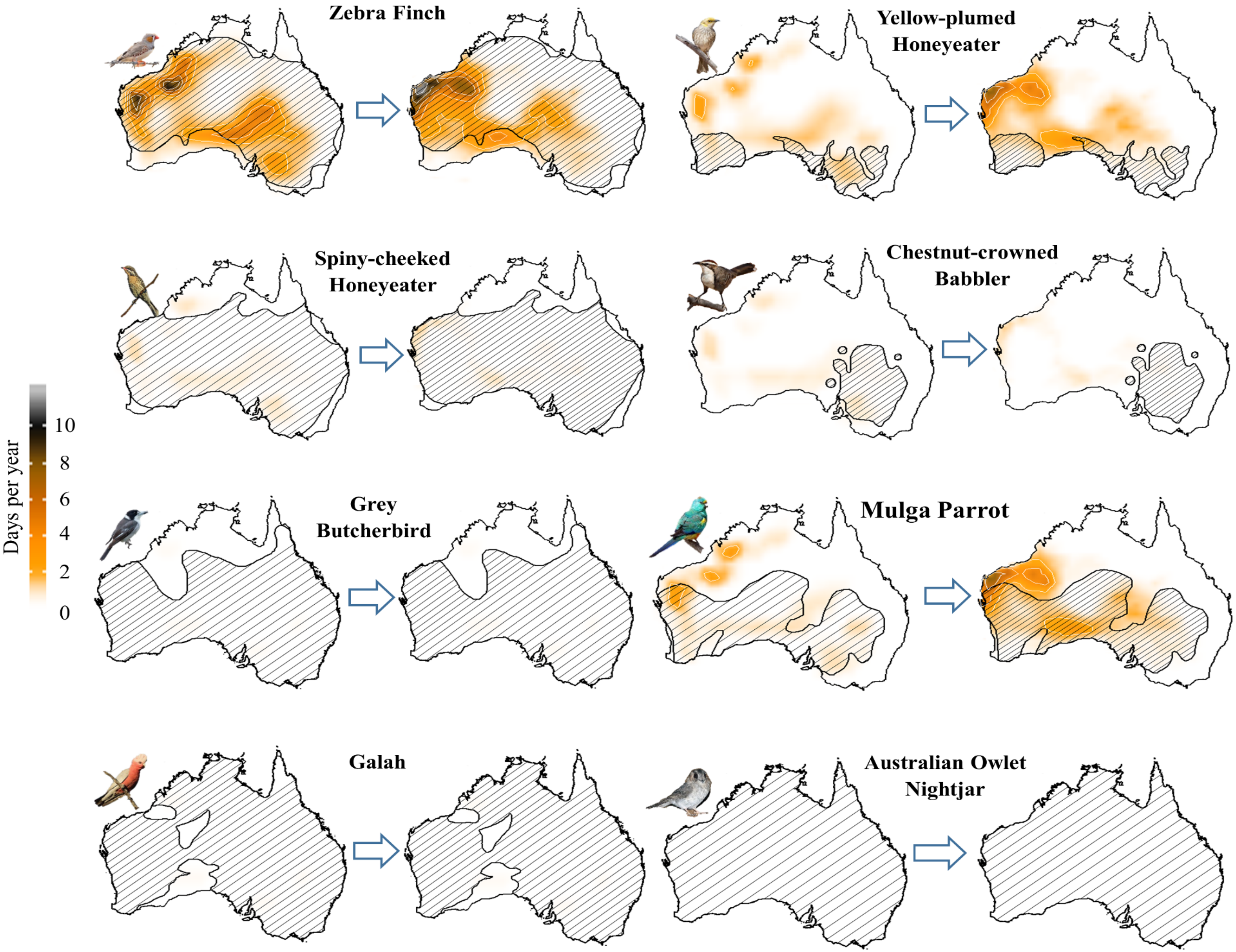

Zebra Finch
Yellow-plumed Honeyeater
Spiny-cheeked Honeyeater
Chestnut-crowned Babbler
Grey Butcherbird
Mulga Parrot
Galah
Australian Owlet Nightjar
Days per year
10
8
6
4
2
0
